# Integrated anthropometric correlates of planned change-of-direction performance (T-test) in male badminton players: a partial least squares regression study

**DOI:** 10.3389/fphys.2026.1844867

**Published:** 2026-06-23

**Authors:** Huiguo Wang, Ziyan Li, Yu Wang, Xianyan Xie, Gaoyuan Yang, Shuqi Qin, Weitong Zhang, Yifeng Li, Tao Chen

**Affiliations:** 1 College of Sport and Health, Guangzhou Sport University, Guangzhou, China; 2 National Academy of Badminton, Guangzhou Sport University, Guangzhou, China

**Keywords:** anthropometry, badminton, male athletes, partial least squares regression, T-test

## Abstract

**Purpose:**

Based on the sport-specific demands of badminton for planned change-of-direction (COD) ability, this study used the T-test to examine the joint association structure between anthropometric indicators and planned COD performance in elite male badminton players, and to identify the high-contribution indicators most closely related to T-test completion time.

**Methods:**

A cross-sectional design was adopted, including 38 elite male badminton players classified as National Level 1 or Level 2 athletes. Anthropometric assessment included length, breadth, and girth variables, together with derived height-normalized ratios/indices and selected segmental proportion indices. Planned COD ability was assessed using the T-test. Exploratory bivariate correlation analyses were first performed. Subsequently, all anthropometric indicators were jointly entered into an integrated partial least squares (PLS) regression model. The optimal number of latent components was determined by cross-validation using the minimum root mean squared error of prediction (RMSEP) criterion. Model performance and the relative contribution of predictors were evaluated using the coefficient of determination (R²), root mean squared error (RMSE), variable importance in projection (VIP), and regression coefficients.

**Results:**

A total of 38 elite male badminton players were included. Correlation analysis showed that hand length (r = -0.448, p = 0.006), sitting height (r = -0.340, *p* = 0.043), Cormic Index (r = -0.389, *p* = 0.019), hand length-to-height ratio (r = -0.482, *p* = 0.003), and forearm length-to-height ratio (r = -0.482, *p* = 0.003) were significantly negatively correlated with T-test completion time. In contrast, lower-limb length (r = 0.365, *p* = 0.029), shank length (r = 0.330, *p* = 0.049), shank length-to-height ratio (r = 0.351, *p* = 0.036), shank-to-thigh index (r = 0.355, *p* = 0.033), Manouvrier’s index (r = 0.384, *p* = 0.021), lower-limb length-to-height ratio (r = 0.384, *p* = 0.021), and brachial–antebrachial index (r = 0.373, *p* = 0.025) were significantly positively correlated with T-test completion time. Further multivariable analysis using an integrated partial least squares (PLS) model demonstrated that the three-component solution provided the best predictive performance, with the lowest cross-validated RMSEP (0.784), an R² of 0.65, and an RMSE of 0.599. VIP analysis showed that the anthropometric information most relevant to T-test completion time was concentrated in a limited subset of variables, particularly hand length-to-height ratio, forearm length-to-height ratio, lower-limb length, lower-limb length-to-height ratio, Manouvrier’s index, shank length, forearm length, hip breadth-to-height ratio, hip breadth, brachial–antebrachial index, and hand shape index. Regression coefficients further indicated that the retained predictors were associated with T-test performance in different directions, rather than contributing uniformly, suggesting a multidimensional structural pattern underlying planned change-of-direction performance.

**Conclusion:**

The present study suggests that T-test performance in elite male badminton players cannot be adequately explained by any single anthropometric indicator alone, but is more likely associated with an integrated morphological profile composed of a limited number of high-contribution variables. The anthropometric information represented in planned change-of-direction performance was mainly concentrated in indicators related to distal upper-limb proportions, lower-limb structural proportions, and segmental proportional configuration. These findings provide a preliminary morphological basis for athlete profiling and training monitoring in badminton. However, given the cross-sectional nature of the study, the observed associations should not be interpreted as causal, and further validation in larger and prospective studies is warranted.

## 1 Introduction

Badminton is a high-intensity intermittent sport in which abrupt stops, rapid re-orientations, and multidirectional displacements occur frequently within rallies (Cabello Manrique and González-Badillo, 2003; Phomsoupha and Laffaye, 2015). Consequently, athletes are required to repeatedly perform rapid kinetic transitions—comprising deceleration, directional reorientation, and re-acceleration—over extremely short time intervals. Match-play analyses have demonstrated that both rally duration and inter-rally recovery operate on a second-level scale; these repeated high-intensity exchanges persist throughout the match, imposing substantial braking and propulsive loads on the locomotor system (Phomsoupha and Laffaye, 2015).Therefore, rather than relying solely on linear sprint speed or general endurance, the successful execution of key on-court actions hinges more on an athlete’s ability to change direction efficiently within limited space and rapidly restore propulsion. Accordingly, change-of-direction (COD) performance is recognized as a fundamental physical determinant underpinning badminton-specific movement efficiency and competitive performance (Chaouachi et al., 2012; Phomsoupha and Laffaye, 2015; Phomsoupha and Laffaye, 2020).

It should be emphasized that change-of-direction (COD) and agility are conceptually distinct constructs. Agility is commonly defined as a rapid, whole-body movement involving a change in velocity or direction in response to an external stimulus, encompassing processes such as stimulus identification, response selection, and movement execution (Sheppard and Young, 2006; Li and Ding, 2021; Young et al., 2021). In contrast, COD refers specifically to the capacity to execute pre-planned directional changes along a predetermined path or toward a defined movement target (i.e., planned COD), primarily reflecting motor-control components such as braking, postural control, and re-acceleration (Brughelli et al., 2008; Young et al., 2021). Therefore, the T-test is essentially a planned COD assessment performed under a preset movement pattern. Its primary focus is the speed of directional change during multidirectional displacement tasks, and it is well suited to reflecting the capacity to accelerate, decelerate, and transition between directions during forward sprinting, lateral shuffling, and backpedaling. However, it does not incorporate the perceptual–cognitive component involved in responding to external stimuli.

From a biomechanical perspective, high-level change-of-direction (COD) performance depends on three interrelated key processes. First, sufficient braking impulse must be generated rapidly before directional reorientation to reduce momentum. Second, lowering and stabilizing the center of mass (CoM) through trunk and pelvic control is required to enhance postural controllability during the turning phase. Third, after the movement direction has been restructured, the ground reaction force must be rapidly redirected to achieve effective re-acceleration (Dos'Santos et al., 2018; Harper et al., 2022). Related studies have shown that force–impulse characteristics during the braking and propulsive phases, contact time, and kinetic differences across COD tasks involving multiple angles all significantly influence performance outcomes such as completion time and take-off velocity (Dos'Santos et al., 2018; Harper et al., 2022). These findings suggest that COD should be understood as an integrated expression of the continuous “braking–turning–propulsion” sequence, rather than as a single speed-related ability.

Within the aforementioned biomechanical processes, anthropometric characteristics may affect COD performance through biomechanical pathways such as moment arms, moment of inertia, and mass distribution, thereby altering energy dissipation and re-acceleration efficiency (Negra et al., 2023). In particular, proportional indices (e.g., segment length ratios and trunk proportions) may better characterize the structure–function relationship (Sammoud et al., 2018). Previous studies have shown that trunk control, center of mass (CoM) velocity, the distribution of braking and propulsive forces, and lower-limb kinematic characteristics during directional changes are all closely associated with performance. Furthermore, anthropometric evidence suggests that structural characteristics such as sitting height and lower-limb length are related to COD performance. In light of these findings, it may be hypothesized that the relative proportions of the trunk and lower limbs influence movement organization and re-acceleration efficiency during the turning phase by affecting CoM regulation, braking posture, and segmental moment of inertia (Sasaki et al., 2011; Dos'Santos et al., 2018). Although associations between anthropometric characteristics and COD performance have been reported across different ball sports, the current evidence remains limited in three main respects. First, existing studies have focused predominantly on sports such as soccer and basketball, whereas badminton is characterized by more pronounced sport-specific movement demands, including short-duration high-intensity actions, frequent anterior–posterior transitions, and rapid lateral displacements. Therefore, its structure–function relationship may not necessarily be consistent with that observed in other ball sports (Phomsoupha and Laffaye, 2015; Barrera-Dominguez et al., 2020; Negra et al., 2023). Second, previous research has largely concentrated on absolute indicators such as height, lower-limb length, body mass, and body composition. Although a small number of studies have begun to incorporate body shape or allometric indices, the relative contribution of proportional structure within the same multivariable framework remains insufficiently elucidated (Silva et al., 2016; Giuriato et al., 2021). Third, anthropometric variables are typically highly intercorrelated. If analysis relies solely on bivariate correlations, superficial associations driven by collinearity may be misinterpreted as independent effects. Therefore, it is necessary to account for the correlation structure among variables within a multivariable modeling framework in order to identify key indicators (Porter et al., 2025).

Against this background, the present study focused on elite male badminton athletes and examined planned change-of-direction performance as assessed by the T-test. To move beyond fragmented interpretations based on isolated variables, this study incorporated anthropometric indicators into a unified multivariable framework and used partial least squares (PLS) regression to evaluate their joint association with T-test completion time. Given the substantial intercorrelations commonly observed among anthropometric measures, this approach was intended to identify the indicators with the greatest contribution to planned COD performance and to clarify the overall morphological profile underlying inter-individual differences in T-test ability among elite male badminton players.

Although direct evidence regarding detailed anthropometric determinants of change-of-direction performance in elite badminton players remains limited, several anthropometric regions may be theoretically relevant according to the biomechanical characteristics of planned change-of-direction tasks. In such tasks, athletes are required to rapidly decelerate, stabilize the body, reposition the center of mass, and re-accelerate in a new direction. Therefore, lower-limb structural dimensions and segmental proportions may be related to braking and re-acceleration mechanics, whereas trunk–leg proportion may influence body control and center-of-mass regulation during directional transitions. In addition, selected breadth-related indicators, such as hip breadth or breadth-to-height ratios, may reflect aspects of transverse body morphology that are potentially relevant to support stability and movement control. Given the limited direct evidence in badminton-specific populations, these variables were examined within an exploratory multivariable framework rather than being treated as isolated causal predictors.

Accordingly, the present study was guided by two working hypotheses. First, anthropometric indicators would exhibit a meaningful multivariable association structure with T-test performance. Second, the contributions of these indicators would be unevenly distributed, with only a limited subset showing relatively greater importance within the integrated model.

## 2 Methods

### 2.1 Participants

This study utilized a cross-sectional design. A total of 38 male badminton athletes (National Level 1 and Level 2) were recruited, all of whom were right-handed players. The participants were aged 20.0 ± 4.0 years and had accumulated 10.50 ± 2.52 years of systematic training experience. All participants were in a regular training phase, with a training frequency of at least four sessions per week and a training duration of approximately 2–3 hours per session.

The inclusion criteria were as follows: (1) no acute lower-limb injuries affecting training or competition within the previous 6 months (e.g., ankle sprains, acute knee or hip pain); (2) no history of major surgery or chronic diseases; and (3) not being in a rehabilitation or sport-specific recovery phase prior to testing. Exclusion criteria included the occurrence of pain or discomfort during testing, or an inability to complete the testing protocol.

All participants provided written informed consent prior to participation. This study was approved by the Ethics Committee of Guangzhou Sport University (Approval No. 2025LCLL-064) and was conducted in accordance with the Declaration of Helsinki.

### 2.2 Experimental design

All measurements in this study were completed within the same training cycle. Testing was conducted in a standard indoor badminton hall, with the ambient temperature maintained at 22–25 °C. To minimize the potential influence of circadian rhythms on physical performance, all assessments were scheduled between 09:00 and 11:00 a.m.

The testing protocol was administered in a fixed order: anthropometric assessments were performed first, followed by a 10-min passive rest period, and finally the completion of the T-test. To reduce the potential interference of recent training loads on performance, participants were instructed to refrain from high-intensity training activities for 48 h prior to testing. On the testing day, a standardized dynamic warm-up was completed before formal assessment. This included 5 min of low-intensity jogging, dynamic stretching, and two submaximal change-of-direction practice runs, ensuring that participants achieved an optimal physiological and neuromuscular state prior to formal testing.

To ensure measurement reliability and reproducibility, both the anthropometric variables and T-test completion time were assessed in triplicate (three times). The mean value of the three trials for each variable was calculated and used for subsequent statistical analyses.

### 2.3 Anthropometric measurements

Anthropometric measurements were performed in accordance with the standardized procedures of the International Society for the Advancement of Kinanthropometry (ISAK) and were completed by the same trained assessor to minimize inter-rater variability. The measurement instruments included a stadiometer (measured to the nearest 0.1 cm), an electronic scale (to the nearest 0.1 kg), sliding anthropometric calipers, and a flexible measuring tape (to the nearest 0.1 cm). All measurements were obtained with the participants barefoot and dressed in light sportswear.

The anthropometric variables were classified into three categories: (1) length variables, including upper-limb length, upper-arm length, forearm length, hand length, lower-limb length (and its segmental components, including thigh length, shank length, and Achilles tendon length), and sitting height; (2) breadth variables, including biacromial breadth, pelvic breadth, and hip breadth; and (3) girth variables, including upper-arm girth, thigh girth, and calf girth, each measured under both relaxed and maximal contraction conditions. For bilateral variables, measurements were obtained on both the left and right sides. Each side was measured three times, and the mean of the three trials was used for subsequent statistical analysis. Detailed definitions and calculation methods of all anthropometric variables are provided in **Supplementary Material 1**.

To control for the potential influence of between-subject differences in body size, derived anthropometric ratios/indices normalized to height, together with selected segmental proportion indices (e.g., shank length-to-thigh length ratio and brachial–antebrachial index), were further calculated. All anthropometric indicators were then jointly considered as candidate predictors in the subsequent multivariable modeling procedure. For bilateral variables, the mean of the left and right sides was calculated and entered into the statistical models.

Test-retest reliability was assessed using a two-way mixed model based on the three repeated measurements. The Intraclass Correlation Coefficients (ICCs) for all anthropometric variables ranged from 0.94 to 0.99 (95% CI: 0.89–1.00), indicating excellent reliability across all measured parameters.

### 2.4 T-test

**Figure 1:**
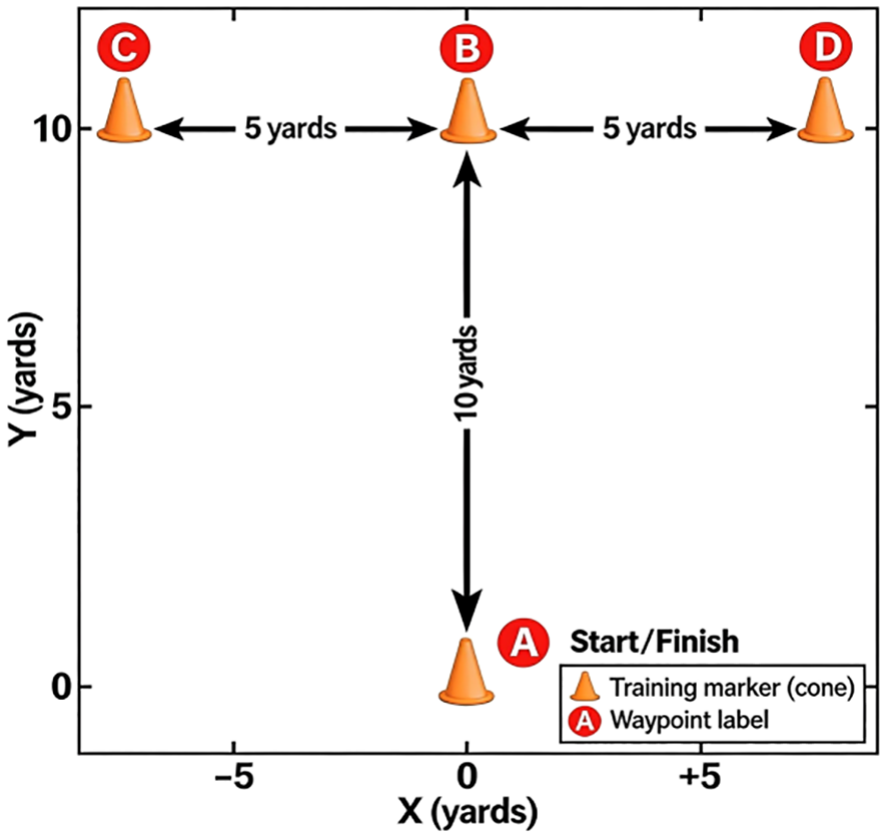
Schematic representation of the agility T-test protocol (adapted from Pauole et al., 2000).

The T-test was conducted in accordance with the standard protocol (Negra et al., 2023). The testing area was arranged in a “T” configuration, with a longitudinal distance of 9.14 m from the starting point (A) to the midpoint (B), and a lateral distance of 4.57 m from point B to each of the two side cones (C and D). The schematic representation of the T-test protocol is shown in **Figure 1**.

At the start of the test, each participant stood behind point A. Once the trial began, the participant was required to complete the following sequence: (1) a forward sprint from A to B; (2) a left lateral shuffle from B to C; (3) a right lateral shuffle from C to D; (4) a left lateral shuffle from D to B; and (5) a backpedal from B to A. Throughout the test, participants were required to face forward and to touch the base of each cone by hand at the change-of-direction points. Trials were considered invalid if a crossover step was used or if the participant failed to touch the base of the cone.

Performance time was recorded using an electronic timing system to the nearest 0.01 s. Each participant completed three valid trials, separated by a 10-min rest interval, and the mean value was used for subsequent analysis.

### 2.5 Statistical analysis

All statistical analyses were performed using SPSS 26.0 and R version 4.5.2. Continuous variables are presented as mean ± standard deviation (SD). Normality was assessed using the Shapiro–Wilk test. For exploratory bivariate analyses, Pearson’s correlation was used for normally distributed variables and Spearman’s rank correlation was applied otherwise. Statistical significance was set at α = 0.05 (two-tailed). Because of the relatively limited sample size and the large number of anthropometric variables, these bivariate analyses were considered exploratory and were not used for variable selection in the multivariable modeling procedure.

For multivariable modeling, only male athletes were included, and T-test completion time was specified as the dependent variable. All numeric anthropometric variables were initially entered as candidate predictors. Missing data were handled using complete-case analysis, and zero-variance predictors were removed before model fitting. To avoid computational issues caused by variable names containing Chinese characters or special symbols, predictor names were temporarily recoded during model iteration and restored to the original labels for final reporting.

Partial least squares (PLS) regression was used to evaluate the integrated association structure between anthropometric indicators and T-test completion time. PLS regression was selected because the anthropometric predictors were relatively numerous and intercorrelated. Modeling was performed using the plsr() function in the R package pls. All predictors were standardized before analysis (scale = TRUE). In the primary PLS analysis, five-fold cross-validation was used to evaluate model performance and to select the optimal number of latent components according to the minimum cross-validated root mean squared error of prediction (RMSEP). Up to 10 latent components were considered in each candidate model, with the actual maximum constrained by the number of predictors and sample size.

Variable selection was conducted using a VIP-based recursive backward elimination procedure. After each PLS model was fitted, variable importance in projection (VIP) scores were calculated, the predictor with the lowest VIP was removed, and the model was refitted using the remaining predictors. This process continued until either the number of retained predictors reached the predefined lower limit (min_vars = 5) or the minimum VIP value reached the predefined threshold (vip_stop = 0.80). Throughout the iterative process, the number of retained predictors, optimal number of latent components, RMSEP, coefficient of determination (R²), root mean squared error (RMSE), minimum VIP value, and removed predictor were recorded. The final model was defined as the model with the lowest cross-validated RMSEP across the entire recursive selection process (Fernández Pierna et al., 2009; Mevik and Wehrens, 2015).

For the final model, VIP scores were used to assess the relative contribution of retained predictors within the PLS latent-component framework, and PLS regression coefficients were used to describe the direction of association with T-test completion time. A negative coefficient indicated that a higher predictor value tended to be associated with a shorter T-test completion time, whereas a positive coefficient indicated an association with a longer completion time. Model performance was evaluated using R², RMSE, and cross-validated RMSEP, and visualized using VIP ranking plots, RMSEP profile plots, and observed-versus-predicted scatter plots.

To further evaluate model robustness, three additional internal validation procedures were performed. First, repeated five-fold cross-validation was conducted 100 times. In each repetition, the sample was randomly divided into five folds; four folds were used for model training and the remaining fold was used for testing. Within each training set, the optimal number of latent components was selected according to the minimum RMSEP criterion. The mean and standard deviation of R², Q², RMSE, and MAE across the 100 repetitions were reported.

Second, bootstrap resampling with 1,000 iterations was used to assess the stability of VIP rankings and regression coefficient directions. In each bootstrap sample, the PLS model was refitted, the optimal number of latent components was reselected, and VIP scores and regression coefficients were recalculated. Variables with high mean VIP values, high VIP ≥ 1 frequency, and consistent coefficient directions were considered relatively more stable within the present exploratory model.

Third, permutation testing with 1,000 iterations was performed to assess whether the observed model performance exceeded chance-level prediction. The dependent variable was randomly permuted while the predictor matrix was kept unchanged, and the complete PLS modeling procedure was repeated for each permuted dataset. Empirical p values were calculated by comparing the observed RMSEP, R², and Q² with their corresponding permutation distributions.

Given the relatively small sample size and the number of candidate anthropometric predictors, the PLS model was interpreted as an exploratory latent-structure approach rather than a confirmatory or externally validated prediction model. The stability and generalizability of the retained predictors require further validation in larger independent samples.

## 3 Results

### 3.1 Descriptive statistics

**Table 1** summarizes the anthropometric characteristics of the 38 elite male badminton players. Continuous variables are expressed as mean ± standard deviation (SD), together with their respective minimum and maximum values. Prior to the subsequent inferential analyses, normality was assessed for all variables using the Shapiro–Wilk test, and no significant deviations from a normal distribution were observed (p > 0.05).

**Table 1:** Descriptive statistics of participant characteristics and key anthropometric variables (N = 38).

Variable	Minimum	Maximum	Mean	SD
body mass	55.9	93	69.9	8.34
Height	163.2	189	176.66	6.33
arm span	161	189.9	175.57	7.43
sitting height	72.2	106.6	97.57	6.89
neck length	8	12.5	9.58	1.06
hand breadth	5.25	10.6	8.92	1.03
biacromial breadth	36.5	49.8	40.87	2.97
pelvic breadth	21	27	24.06	1.65
hip breadth	24	35	29.47	2.75
neck circumference	23.1	41	35.81	2.75
chest circumference	78.4	106	90.11	6.13
waist circumference	66	93.7	77.31	6.12
hip circumference	85.5	113.7	96.31	5.81
upper-arm circumference (flexed)	26	38.55	29.97	2.97
upper-arm circumference (relaxed)	22.5	35.8	26.77	3.1
forearm circumference	22.5	30.25	25.39	1.62
upper-limb length	68.25	85.65	76.2	3.74
upper-arm length	27	36.85	30.9	2.16
forearm length	21.4	29.7	26.28	1.75
palm length	8	14.5	10.71	1.05
hand length	17.5	22.15	19.01	0.92
lower-limb length	68.4	104.35	79.13	7.97
thigh length	33.5	45.95	39.43	3.16
shank length	34.9	46	40.67	2.53
achilles tendon length	22.25	34.25	26.95	2.47
thigh circumference (contracted)	45.5	68.5	57.01	4.97
thigh circumference (relaxed)	44.35	67	56.07	5.01
calf circumference (contracted)	34.7	43.25	38.16	1.91
calf circumference (relaxed)	34	41.65	37.68	1.95
ankle circumference	19.8	25.5	22.56	1.28
hand length-to-height ratio	0.099	0.121	0.108	0.005
lower-limb length-to-height ratio	0.405	0.587	0.448	0.038
upper-limb length-to-height ratio	0.404	0.469	0.431	0.014
upper-arm length-to-height ratio	0.156	0.198	0.175	0.009
forearm length-to-height ratio	0.126	0.165	0.147	0.008
palm length-to-height ratio	0.047	0.079	0.061	0.005
thigh length-to-height ratio	0.192	0.25	0.223	0.015
shank length-to-height ratio	0.205	0.258	0.23	0.011
achilles tendon length-to-height ratio	0.122	0.188	0.153	0.013
hand breadth-to-height ratio	0.031	0.059	0.05	0.005
biacromial breadth-to-height ratio	0.203	0.272	0.231	0.016
pelvic breadth-to-height ratio	0.115	0.157	0.136	0.01
hip breadth-to-height ratio	0.138	0.2	0.167	0.016
neck length-to-height ratio	0.044	0.072	0.054	0.007
arm span-to-height ratio	0.947	1.038	0.994	0.022
Cormic Index	0.414	0.598	0.553	0.038
Manouvrier’s index	0.677	1.419	0.82	0.154
brachial–antebrachial index	0.705	1.007	0.853	0.064
shank-to-thigh index	0.867	1.322	1.037	0.103
hand shape index	0.282	0.561	0.47	0.055
thigh-to-calf girth ratio (contracted)	1.247	1.791	1.493	0.097
thigh-to-calf girth ratio (relaxed)	1.232	1.759	1.487	0.097
achilles tendon length index	0.515	0.834	0.664	0.063
calf morphology index (contracted)	1.434	1.8	1.694	0.074
calf morphology index (relaxed)	1.415	1.787	1.673	0.078

Data are presented as minimum, maximum, mean, and standard deviation (N = 38). Body mass is expressed in kg, whereas all other anthropometric length, breadth, and girth variables are expressed in cm. All “-to-height ratio” variables and all indices are dimensionless ratios. lower-limb length was defined as the vertical distance from the gluteal fold to the ground. Manouvrier’s index (also referred to here as the skeletal index) = lower-limb length/sitting height; brachial–antebrachial index = forearm length/upper-arm length; shank-to-thigh index = shank length/thigh length; hand shape index = hand breadth/hand length; Achilles tendon length index = Achilles tendon length/shank length; calf morphology index (contracted/relaxed) = calf circumference (contracted/relaxed)/ankle circumference; thigh-to-calf girth ratio (contracted/relaxed) = thigh circumference (contracted/relaxed)/calf circumference (contracted/relaxed).

### 3.2 Bivariate correlation analysis between anthropometric variables and T-test completion time

**Table 2** presents the correlations between anthropometric variables and T-test completion time. The results showed that hand length (r = -0.448, *p* = 0.006), sitting height (r = -0.340, *p* = 0.043), Cormic Index (r = -0.389, *p* = 0.019), hand length-to-height ratio (r = -0.482, *p* = 0.003), and forearm length-to-height ratio (r = -0.482, *p* = 0.003) were significantly negatively correlated with T-test completion time. In contrast, lower-limb length (r = 0.365, *p* = 0.029), shank length (r = 0.330, *p* = 0.049), shank length-to-height ratio (r = 0.351, *p* = 0.036), shank-to-thigh index (r = 0.355, *p* = 0.033), Manouvrier’s index (r = 0.384, *p* = 0.021), lower-limb length-to-height ratio (r = 0.384, *p* = 0.021), and brachial–antebrachial index (r = 0.373, *p* = 0.025) were significantly positively correlated with T-test completion time. The absolute magnitudes of the correlation coefficients ranged from 0.33 to 0.48, indicating associations of moderate magnitude in this exploratory analysis. However, these bivariate associations were exploratory and should be interpreted together with the subsequent multivariable PLS results.

**Table 2:** Exploratory correlation matrix between anthropometric variables and t-test completion time.

Dimension	Variable	Correlation coefficient (r)	p
Length	Sitting height	-0.34	0.043
	hand length	-0.448	0.006
	lower-limb length	0.365	0.029
	Shank length	0.33	0.049
Relative length	hand length-to-height ratio	-0.482	0.003
	lower-limb length-to-height ratio	0.384	0.021
	Forearm length-to-height ratio	-0.482	0.003
	Shank length-to-height ratio	0.351	0.036
	Cormic Index	-0.389	0.019
	Manouvrier’s index	0.384	0.021
	brachial–antebrachial index	0.373	0.025
	Shank-to-thigh index	0.355	0.033

The correlation coefficients reported in the table are Pearson’s *r*, and *p* values were derived from two-tailed tests. T-test completion time was expressed as completion time (s), with lower values indicating better planned change-of-direction performance. Accordingly, *r* < 0 indicates that a larger anthropometric value was associated with a shorter T-test time (better performance), whereas *r* > 0 indicates that a larger anthropometric value was associated with a longer T-test time (poorer performance). Statistical significance was set at *p* < 0.05.

### 3.3 Integrated PLS model of anthropometric predictors of T-test performance

#### 3.3.1 Model performance and optimal number of latent components

**Figure 2:**
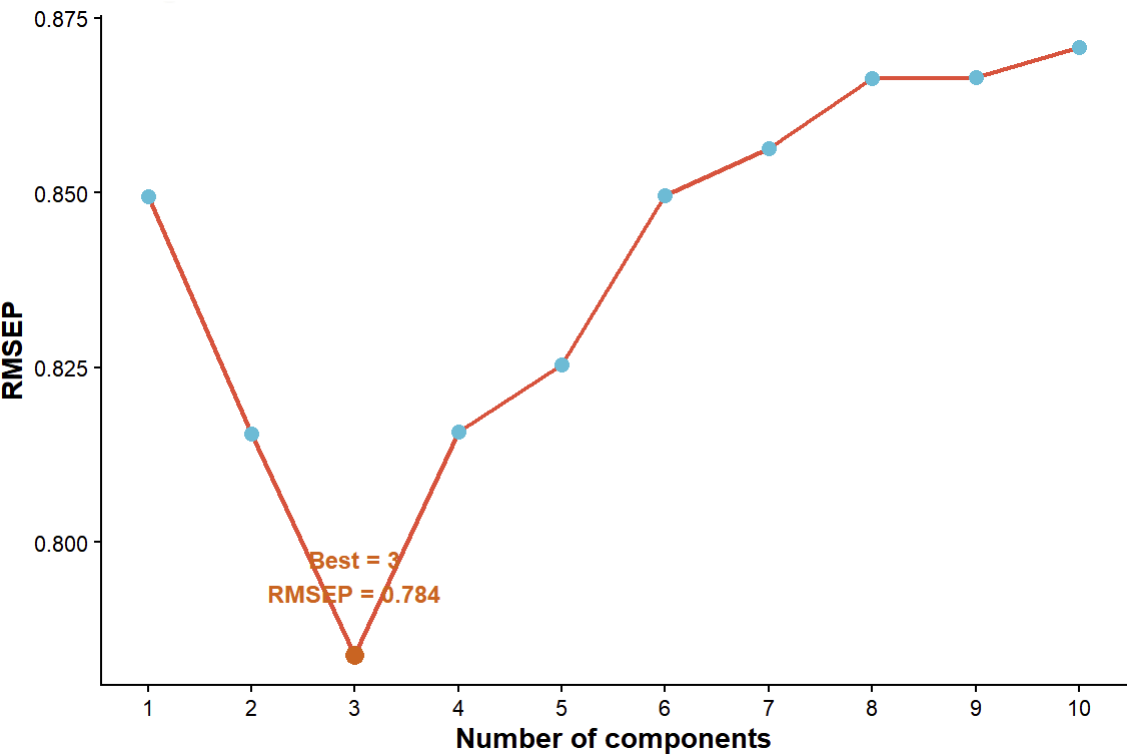
Cross-validated RMSEP across latent components.

To examine the joint association between anthropometric indicators and T-test completion time, an integrated partial least squares (PLS) regression model was established. As shown in **Table 3** and **Figure 2**, the cross-validated RMSEP decreased from 0.849 in the one-component model to 0.815 in the two-component model and reached its minimum value of 0.784 in the three-component model. Thereafter, RMSEP increased as additional latent components were included. Based on the minimum-RMSEP criterion, the three-component solution was selected as the optimal model. The three-component PLS model explained 65.0% of the variance in T-test completion time and yielded an apparent RMSE of 0.599 s, indicating acceptable apparent model fit within the present sample.

**Table 3:** Cross-validated model performance of the integrated partial least squares (PLS) model for T-test completion time.

Number of components	RMSEP
1	0.849380318
2	0.815472227
3	0.783891426
4	0.815752999
5	0.825396296
6	0.849615105
7	0.856288575
8	0.86635859
9	0.86653681
10	0.870832327
Model summary: Optimal number of components = 3; R² = 0.65; RMSE = 0.599.	

The optimal number of latent components was determined according to the minimum cross-validated RMSEP. Model fit was evaluated using R² and RMSE.

#### 3.3.2 Variable importance in the integrated model

The relative importance of retained anthropometric predictors in the integrated PLS model was assessed using VIP scores. As presented in **Table 4**, VIP values were unevenly distributed across variables. The highest contributions were observed for hand length-to-height ratio and forearm length-to-height ratio (both VIP = 1.218), followed by lower-limb length (VIP = 1.138), lower-limb length-to-height ratio (VIP = 1.107), Manouvrier’s index (VIP = 1.102), shank length (VIP = 1.077), forearm length (VIP = 1.046), hip breadth-to-height ratio (VIP = 1.010), hip breadth (VIP = 1.009), brachial–antebrachial index (VIP = 1.007), and hand shape index (VIP = 1.007). These findings indicate that the anthropometric information most relevant to T-test completion time was primarily concentrated in a subset of indicators reflecting distal upper-limb proportion, lower-limb structural proportion, and transverse hip morphology. The remaining variables showed VIP values below 1.0, suggesting comparatively smaller contributions to the latent predictive structure.

**Table 4:** Variable importance in projection (VIP) scores of anthropometric predictors in the integrated PLS model.

Rank	Predictor	VIP
1	hand length-to-height ratio	1.217721645
2	forearm length-to-height ratio	1.217721645
3	lower-limb length	1.138284019
4	lower-limb length-to-height ratio	1.107309387
5	Manouvrier’s index	1.102342203
6	shank length	1.07697624
7	forearm length	1.045877007
8	hip breadth-to-height ratio	1.009851603
9	hip breadth	1.008911892
10	brachial–antebrachial index	1.007182385
11	hand shape index	1.007112012
12	shank-to-thigh index	0.958889057
13	shank length-to-height ratio	0.933760026
14	forearm circumference	0.878816823
15	hand breadth	0.878768793
16	sitting height	0.840196823
17	relaxed thigh circumference	0.839219052
18	arm span-to-height ratio	0.813062117
19	hand breadth-to-height ratio	0.754967003

A VIP value > 1.0 was considered indicative of relatively high contribution to the PLS model.

#### 3.3.3 Directional contributions of predictors

**Figure 3:**
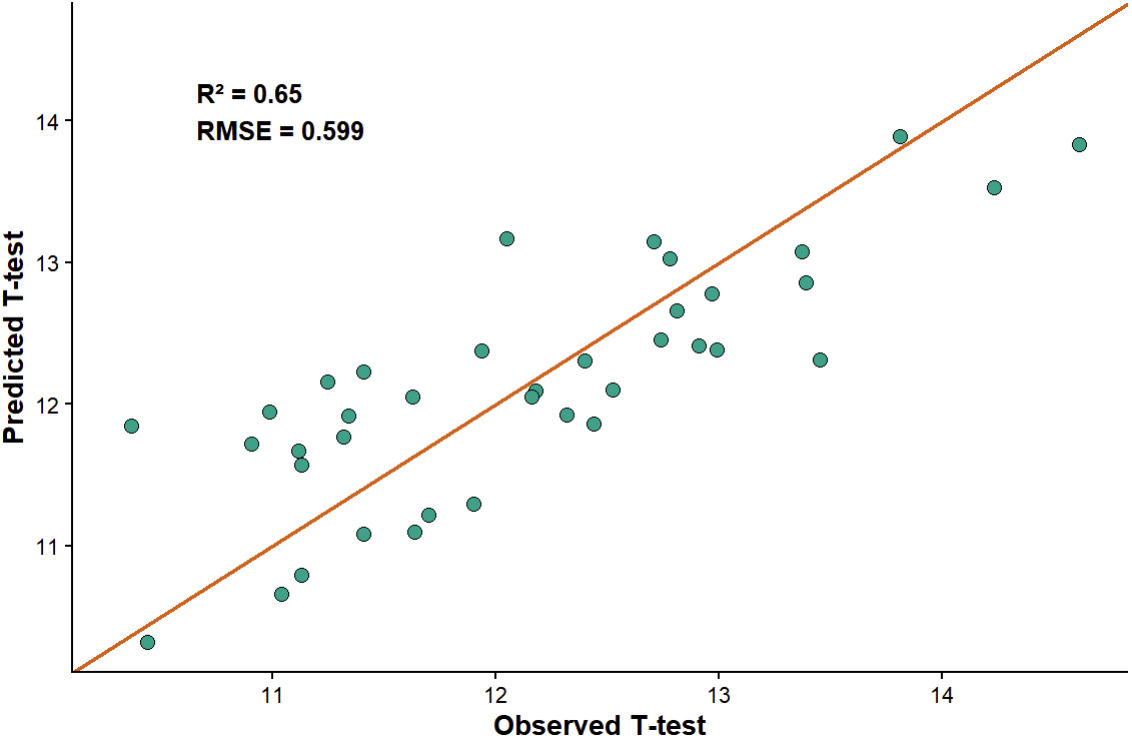
Observed versus predicted T-test values in the integrated PLS model.

The directional contribution pattern of the predictors within the latent component framework is summarized in **Table 5**, and the overall correspondence between observed and model-estimated T-test values is illustrated in **Figure 3**. Within the integrated PLS model, brachial–antebrachial index, hip breadth-to-height ratio, hip breadth, shank-to-thigh index, Manouvrier’s index, and lower-limb length-to-height ratio showed relatively larger positive coefficients, indicating that higher values of these indicators tended to be associated with longer T-test completion time. By contrast, arm span-to-height ratio, hand length-to-height ratio, forearm length-to-height ratio, and sitting height showed the largest negative coefficients, suggesting associations with shorter completion time. The remaining retained variables showed comparatively small coefficients in either direction. Taken together, these findings indicate that the retained anthropometric predictors were linked to T-test performance in different directions within the latent component framework, supporting the view that planned change-of-direction performance is associated with an integrated structural profile rather than any single isolated indicator.

**Table 5:** Regression coefficients and directional contributions of anthropometric predictors in the integrated PLS model.

Predictor	Coefficient	Direction
brachial–antebrachial index	0.217	Positive
arm span-to-height ratio	-0.19	Negative
hip breadth-to-height ratio	0.183	Positive
hand length-to-height ratio	-0.18	Negative
forearm length-to-height ratio	-0.18	Negative
hip breadth	0.179	Positive
shank-to-thigh index	0.154	Positive
sitting height	-0.14	Negative
Manouvrier’s index	0.137	Positive
lower-limb length-to-height ratio	0.125	Positive
shank length-to-height ratio	0.096	Positive
lower-limb length	0.087	Positive
forearm circumference	0.081	Positive
relaxed thigh circumference	0.056	Positive
shank length	0.052	Positive
hand shape index	0.049	Positive
forearm length	0.048	Positive
hand breadth	-0.019	Negative
hand breadth-to-height ratio	-0.01	Negative

Positive coefficients indicate that higher predictor values were associated with longer T-test completion time within the PLS latent component framework, whereas negative coefficients indicate an association with shorter T-test completion time.

#### 3.3.4 Model robustness and internal validation analyses

To further evaluate model robustness, repeated five-fold cross-validation, bootstrap stability analysis, and permutation testing were additionally performed. Repeated five-fold cross-validation across 100 repetitions yielded a mean R² of 0.388 ± 0.055, a mean Q² of 0.351 ± 0.072, and a mean RMSE of 0.816 ± 0.044 s, indicating moderate internal predictive performance, although the apparent model fit was likely optimistic.

Bootstrap stability analysis showed that hand length-to-height ratio demonstrated the most stable contribution, with a mean VIP of 1.238 and a VIP ≥ 1 frequency of 82.1%. Its coefficient direction was consistently negative across bootstrap samples. Lower-limb length-to-height ratio also showed a mean VIP greater than 1.0 and a stable positive coefficient direction, although its VIP ≥ 1 frequency was lower (66.2%).

Permutation testing further supported the robustness of the model. Within the permutation framework, the observed non-permuted model showed a lower RMSEP than the permuted models (0.785 vs. 1.099, empirical p = 0.001) and a higher R² than the permutation distribution (0.590 vs. 0.141, empirical p = 0.001). These findings suggest that the predictive structure captured by the PLS model was unlikely to be explained by random associations between anthropometric predictors and T-test completion time.

## 4 Discussion

### 4.1 Integrated morphological profile underlying T-test performance

The main finding of this study is that T-test performance in elite male badminton players was not explained by a single anthropometric indicator. Instead, it was associated with a combined morphological profile involving several high-contribution variables. Compared with bivariate correlation or stepwise regression, the PLS model was more suitable for this analysis because it can account for correlations among anthropometric predictors. The results showed that the anthropometric information related to T-test completion time was mainly concentrated in variables reflecting distal upper-limb proportions, lower-limb segmental proportions, and transverse hip morphology. This suggests that planned change-of-direction performance in badminton may be more closely related to body proportion and structural configuration than to any isolated body-size characteristic.

This interpretation is biomechanically plausible. The T-test is a planned change-of-direction task performed along a fixed path. Successful performance requires braking control, center-of-mass regulation, intersegmental coordination, and rapid movement reorganization. Therefore, the most relevant anthropometric characteristics may not be absolute lengths or girths alone. Rather, their functional relevance may depend on how different body segments are proportioned and coordinated during movement.

The direction of the coefficients also requires cautious interpretation. Because the dependent variable was T-test completion time, positive coefficients indicate an association with longer completion time, whereas negative coefficients indicate an association with shorter completion time. However, these coefficients were derived from the PLS latent-component framework. They should therefore not be interpreted as independent causal effects of individual variables. Instead, they reflect the joint contribution of correlated morphological characteristics to planned change-of-direction performance.

Overall, these findings suggest that the relationship between anthropometry and T-test performance is not simply determined by stature, body mass, or a single limb dimension. Rather, it appears to depend on the integrated organization of body proportions. From a practical perspective, this supports the use of anthropometric profiling that includes proportional and segmental indicators, rather than relying only on conventional measures such as height and body mass. Nevertheless, because this study used a cross-sectional design, the identified variables should be interpreted as structural correlates of T-test performance, not as causal determinants or fixed structural advantages.

### 4.2 Distal upper-limb proportional characteristics and T-test performance

The present study showed that several high-contribution predictors were related to distal upper-limb proportions. This suggests that T-test performance may not depend solely on lower-limb structure. Upper-limb configuration, especially in the distal segments, may also contribute to the movement organization required for planned change-of-direction (COD) tasks. Although the T-test is often considered a lower-limb-dominant COD task, rapid deceleration, directional change, and re-acceleration require coordinated control across multiple body segments. In this process, arm swing may help regulate angular momentum, maintain balance, and support trunk reorientation (Koo et al., 2025). Therefore, distal upper-limb proportions may not directly improve propulsive capacity, but they may indirectly affect performance by influencing arm-swing efficiency and coordination during rapid body reorientation.

The upper-limb-related variables did not show a uniform pattern of association. Hand length-to-height ratio and forearm length-to-height ratio were associated with shorter completion times, whereas the brachial index showed a positive regression coefficient. This indicates that the functional role of distal upper-limb structure cannot be explained by a simple “longer is better” or “shorter is better” pattern (Popowczak et al., 2022; Kozlenia et al., 2024). A more plausible explanation is that different upper-limb proportions may influence different task components. These may include trunk lean during marker contact, segmental inertia distribution, and transitional efficiency between successive turns (Pontzer et al., 2009). heir effects are therefore likely to be task-dependent. Because the T-test requires repeated contact with the marker bases (Silva et al., 2013), distal upper-limb reach may be particularly relevant to movement efficiency in this testing context.

Accordingly, the present upper-limb findings suggest that distal upper-limb proportions may form part of the structural organization related to planned COD performance. Their role may be reflected mainly in the modulation of coordination demands during directional transitions, rather than in a direct morphological advantage. However, because upper-limb kinematics and angular momentum were not directly measured in this study, these mechanistic interpretations remain tentative and should be examined in future research.

### 4.3 Lower-limb structural proportions and change-of-direction efficiency

Lower-limb structural proportions were another key dimension related to T-test performance in this study. Variables such as lower-limb length, lower-limb length-to-stature ratio, the Manouvrier index, shank length, and the shank-to-thigh ratio were retained in the final model. This indicates that lower-limb length characteristics and segmental proportions were associated with planned change-of-direction (COD) performance (Chaouachi et al., 2012; Negra et al., 2023). This finding is generally consistent with biomechanical research showing that lower-limb structure may influence not only propulsion, but also braking posture, support strategy, and force redirection efficiency.

Several lower-limb proportional indices were positively associated with T-test completion time. This suggests that, in the present sample, relatively greater lower-limb proportions were not necessarily associated with better performance (Spiteri et al., 2013). Although longer lower limbs may be beneficial in linear sprinting, the T-test places different mechanical demands on athletes. Performance in this short-distance planned COD task depends less on stride length and more on rapid deceleration, lowering the center of mass, and reorienting propulsion within a confined space (Sheppard and Young, 2006; Mulligan et al., 2024). Thus, longer lower-limb structures may provide greater reach, but they may also increase the demands of braking control and postural reorganization during directional transitions.

The retention of shank-related variables further suggests that distal lower-limb configuration may be more relevant than overall lower-limb length alone. The shank segment is involved in foot repositioning, eccentric buffering, and the transition from braking to propulsion. Therefore, variation in this segment may influence movement reorganization during COD tasks. These findings indicate that the key issue is not simply whether longer or shorter lower limbs are more favorable, but how lower-limb proportions are associated with control demands and transition efficiency. Because this study did not include kinetic measures such as force-platform data, joint moments, or center-of-mass trajectories, the underlying mechanical pathways require further investigation.

### 4.4 Transverse hip morphology and segmental configuration in planned COD tasks

In addition to upper- and lower-limb proportions, hip breadth and hip breadth-to-height ratio were retained in the final model. This suggests that transverse hip morphology may be part of the integrated structural profile associated with planned change-of-direction (COD) performance. Compared with longitudinal measures such as stature or segment length, hip breadth has received less attention in COD research. However, in the present study, hip-related variables made relatively high contributions to the model and were positively associated with T-test completion time. This indicates that transverse pelvic morphology may be related to movement efficiency during directional transitions (Straub and Powers, 2022; Kramer and Sylvester, 2023).

This finding is biomechanically plausible. The pelvis links trunk control with lower-limb force transmission. During rapid COD tasks, frontal-plane stability, trunk alignment, and support-foot repositioning all require coordinated control of the pelvic region (Kuhne et al., 2026). A more pronounced transverse hip morphology may alter the position of the center of mass relative to the base of support. It may also increase the postural and coordination demands during deceleration and turning (Ujaković and Šarabon, 2020). Therefore, the present results do not suggest that wider hips are generally disadvantageous. Rather, they indicate that, under the specific task constraints of the T-test, greater hip breadth may be associated with higher demands for postural reorganization.

Importantly, hip-related variables were not retained in isolation. They appeared in the final model together with several upper- and lower-limb proportional indicators. This suggests that the role of hip breadth is embedded within the overall segmental configuration, rather than acting as an independent anatomical determinant of performance. In other words, the functional relevance of transverse hip morphology may depend on its combined relationship with limb proportions and task-specific coordination demands (Smith et al., 2021; Straub and Powers, 2022). This further supports the latent structural pattern identified by the PLS model, in which planned COD performance is associated with a multi-segment and coordinative morphological profile.

### 4.5 Differences between exploratory correlations and multivariable latent-structure results

An important methodological finding of this study is that the exploratory bivariate correlations were not fully consistent with the integrated PLS results. Bivariate correlations can identify simple linear associations between individual anthropometric variables and T-test performance. However, they cannot fully account for the strong intercorrelations among anthropometric indicators. In contrast, the PLS model considers the covariance structure among multiple predictors simultaneously. It is therefore more suitable for identifying variables that carry meaningful information within the overall latent structural pattern (Smith et al., 2021).

Some variables were significant in the bivariate analysis but contributed only weakly to the final model. Conversely, some variables showed weak simple correlations but became important predictors in the multivariable framework. This discrepancy should not be viewed as a contradiction. Instead, it reflects a key feature of anthropometric data: body segments are highly interrelated and do not function independently (Kimura et al., 2021; Sheikhi et al., 2021). Under such conditions, univariable correlations may overestimate the role of some indicators. They may also overlook variables that are meaningful only within a broader structural pattern.

From this perspective, the conclusions of the present study are based not only on pairwise associations, but also on latent structural patterns extracted from the covariation among anthropometric variables. This approach improves the interpretability of the findings and may be useful in sports anthropometric research, especially when sample size is limited, the number of indicators is relatively large, and multicollinearity is present. Nevertheless, PLS is primarily used for structural extraction and association modeling, not for causal identification. Therefore, the retained variables should be interpreted cautiously as structural correlates of T-test performance.

### 4.6 Practical implications for anthropometric evaluation and training monitoring in badminton

From a practical perspective, the present findings offer several implications for anthropometric assessment and sport-specific training monitoring in badminton players. First, the structural information associated with planned change-of-direction (COD) ability does not appear to be primarily reflected in traditional indicators such as stature or body mass, but rather in proportional relationships and segmental configuration. This suggests that if morphological assessment relies solely on absolute body dimensions, potentially meaningful differences related to short-distance COD performance may be difficult to detect fully (Negra et al., 2023). In contrast, proportional indicators such as hand length-to-height ratio, forearm length-to-height ratio, lower-limb length-to-height ratio, the Manouvrier index, and hip breadth-to-height ratio may be more informative as supplementary observational dimensions.

Second, the practical value of these findings for training monitoring lies mainly in explaining inter-individual differences rather than directly informing body-shape intervention. Anthropometric characteristics are generally difficult to modify substantially through training over a short period (Lijewski et al., 2021; Ramirez-Munera et al., 2024), but their relevance lies in helping coaches understand the structural advantages and constraints that different athletes may exhibit during deceleration, directional transition, and re-acceleration. This, in turn, may provide useful background information for individualizing technical and physical training. For example, athletes with more pronounced lower-limb proportions may require greater emphasis on braking stability and center-of-mass control (Sisic et al., 2016), whereas athletes with more pronounced distal upper-limb proportions may display different characteristics in marker-contact transitions and arm-swing organization.

It should be noted that the present study does not support the simple use of these indicators as talent identification criteria or direct predictors of performance. From a practical perspective, coaches and practitioners should interpret anthropometric characteristics as part of an integrated movement-related profile rather than as isolated selection criteria. Distal upper-limb proportions, lower-limb segmental proportions, and transverse hip morphology may help describe inter-individual differences in movement organization during planned COD tasks. However, these indicators should not be used alone to judge athletic potential. Instead, they may provide supplementary information for individualized agility training, movement screening, and technical assessment when combined with performance tests, strength evaluation, and biomechanical observation. Accordingly, the practical significance of this study does not lie in proposing an “ideal body-type template,” but in promoting a shift in badminton anthropometric assessment from static body-shape description toward a more functional role in movement interpretation and training-related explanation.

### 4.7 Limitations and future directions

Although the present study revealed an integrated pattern of association between anthropometric characteristics and T-test performance in elite male badminton players, several limitations should be acknowledged and the findings interpreted with caution. First, the study employed a cross-sectional design; therefore, all results are inherently structural associations and cannot be used to infer causal direction. In addition, the exploratory bivariate correlation analyses involved multiple pairwise tests, and no formal Bonferroni or false discovery rate correction was applied. Thus, the nominally significant bivariate correlations should be interpreted cautiously as preliminary descriptive evidence rather than confirmatory findings. In other words, the present findings indicate only that certain proportional characteristics are associated with planned change-of-direction (COD) performance, but do not demonstrate that these structural features directly cause performance differences. Second, the sample size was relatively limited, and the participants were restricted to elite male badminton players, which to some extent constrains the generalizability of the findings. Whether similar structure–function relationships exist across different competitive levels, between sexes, or even among athletes from different sport-specific backgrounds remains to be further verified. Third, the T-test used in the present study represents a typical planned COD task. The T-test should be interpreted as a standardized assessment of planned COD performance rather than a direct measure of badminton-specific reactive agility. Although it includes forward sprinting, lateral shuffling, directional transitions, and backpedaling, it does not reproduce shuttle-specific footwork patterns, perceptual–cognitive processing, or opponent-induced decision-making demands in real match play. Therefore, the present findings should not be generalized to badminton agility as a whole. Nevertheless, in badminton training practice, planned COD ability can be regarded as a foundational component that precedes more complex unplanned or reactive COD tasks. From this perspective, the T-test remains useful for isolating basic multidirectional locomotor transitions under standardized conditions, although it should be complemented by badminton-specific footwork and reactive agility assessments in future studies. Fourth, several ratio-based anthropometric indicators in the present study were derived from overlapping absolute measurements and shared denominators. Such derived variables may introduce redundancy, ratio inflation, and mathematical coupling within the predictor set. Therefore, these ratio-based indicators should be interpreted as descriptive markers of proportional configuration rather than as independent anatomical determinants. Fifth, the present study included only external anthropometric variables and did not incorporate internal mechanistic measures such as kinematics, kinetics, ground reaction forces, or neuromuscular control. Consequently, explanations for why certain proportional characteristics were associated with better or poorer T-test performance remain largely theoretical. The biomechanical interpretations regarding braking mechanics, center-of-mass control, movement reorganization, or segmental coordination should therefore be regarded as plausible hypotheses rather than direct mechanistic evidence. Seventh, the bivariate correlation analyses were exploratory in nature, and no formal multiple-comparison correction was applied. Therefore, the pairwise correlation results should be interpreted cautiously and should not be regarded as confirmatory evidence. The main conclusions of the present study were based primarily on the integrated PLS model and the subsequent robustness analyses rather than on individual unadjusted correlations. Eighth, the participants were limited to elite male badminton players. Therefore, the generalizability of the findings to female athletes, younger athletes, recreational players, or athletes from other competitive levels remains uncertain. Future studies should include more diverse samples to examine sex-specific and performance-level-specific patterns.

Despite these limitations, the present study still provides clear value. At a minimum, it shows that in badminton-specific research on planned COD, integrated latent-structure analysis can extract morphologically meaningful information with greater explanatory relevance from a large number of anthropometric variables. Future studies should build on this foundation by expanding sample size, including female athletes and groups of different competitive standards, and combining three-dimensional motion capture, kinetic analysis, and electromyographic assessment to systematically clarify the specific mechanical and neuromuscular pathways through which body proportional characteristics influence COD performance in badminton. In addition, future work may consider combining planned COD tests with reactive agility tests in order to distinguish the boundaries of structural influences across different types of movement ability.

The T-test should be interpreted as a standardized assessment of planned COD performance rather than a direct measure of badminton-specific reactive agility. Although it includes forward sprinting, lateral shuffling, and backpedaling, it does not reproduce shuttle-specific footwork patterns, perceptual–cognitive processing, or opponent-induced decision-making demands in real match play. Therefore, the present findings should not be generalized to badminton agility as a whole.

Future studies should integrate force-platform analysis, three-dimensional motion capture, joint kinematics and kinetics, and electromyographic assessment to clarify the mechanical and neuromuscular pathways through which anthropometric proportions may relate to planned COD performance.

## 5 Conclusion

The present study suggests that T-test performance in elite male badminton players cannot be adequately explained by any single anthropometric indicator alone, but is more likely associated with an integrated morphological profile composed of a limited number of high-contribution variables. The anthropometric information represented in planned change-of-direction performance was mainly concentrated in indicators related to distal upper-limb proportions, lower-limb structural proportions, and segmental proportional configuration. These findings provide a preliminary morphological basis for athlete profiling and training monitoring in badminton. However, given the cross-sectional nature of the study, the observed associations should not be interpreted as causal, and further validation in larger and prospective studies is warranted.

## Data Availability

The raw data supporting the conclusions of this article will be made available by the authors, without undue reservation.

## Supplementary material

Supplementary Figure 1 Study design and measurement workflow.
