# Supplementary figures and images for: Integrated anthropometric correlates of planned change-of-direction performance (T-test) in male badminton players: a partial least squares regression study
Source: Front Physiol. 2026 Jun 23;17:1844867. doi: 10.3389/fphys.2026.1844867 (PMC13337512; doi:10.3389/fphys.2026.1844867)

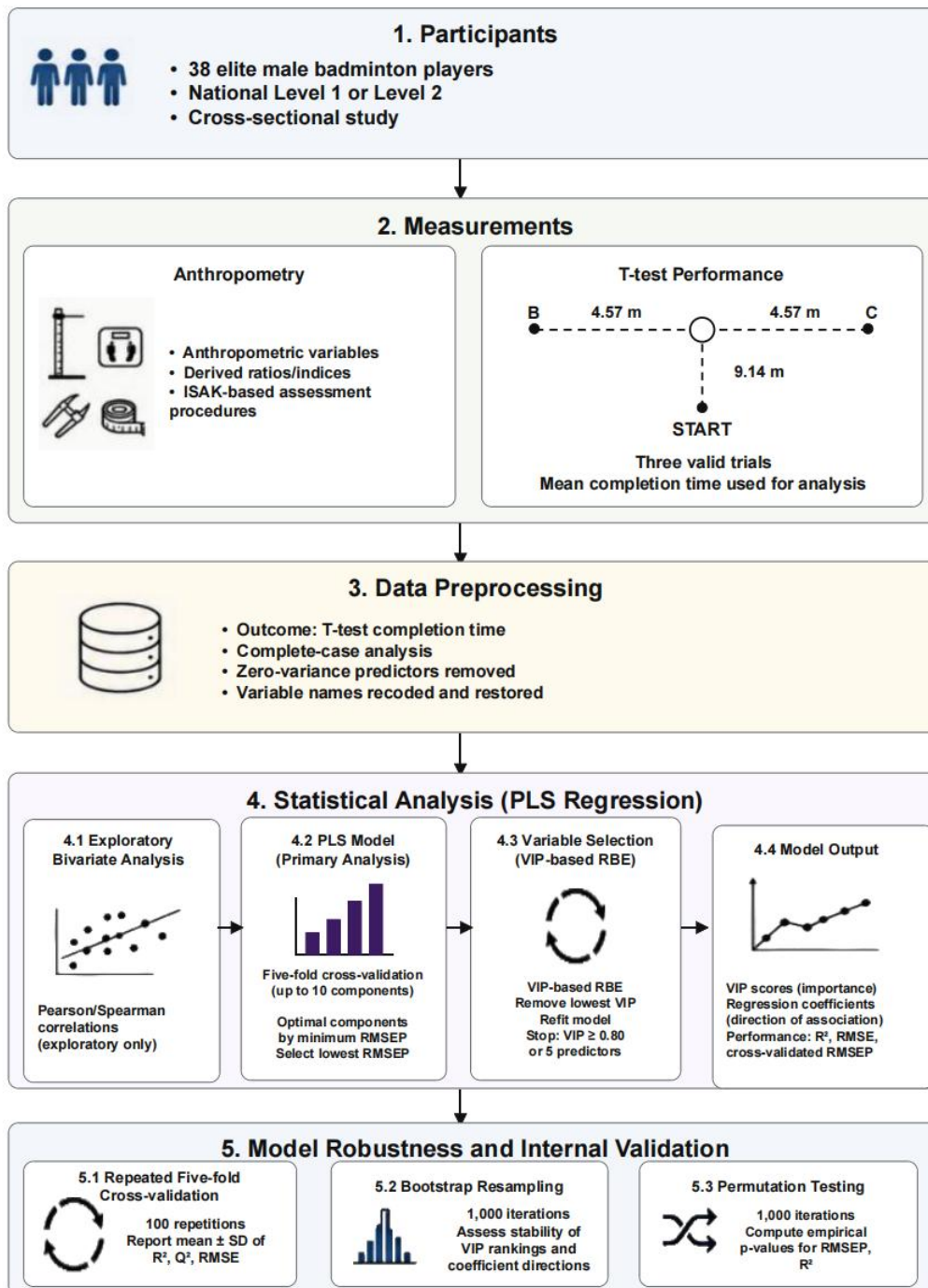

**Supplementary Figure 1.** Study design and measurement workflow

Supplement: Supplementary Figure 1 — Study design and measurement workflow. [file Image1.pdf]
