# Supplementary material for: Integrated anthropometric correlates of planned change-of-direction performance (T-test) in male badminton players: a partial least squares regression study
Source: Front Physiol. 2026 Jun 23;17:1844867. doi: 10.3389/fphys.2026.1844867 (PMC13337512; doi:10.3389/fphys.2026.1844867)
Supplement: Supplementary file 2 [file Presentation1.pdf]

Supplementary Material 1.

Definitions of Anthropometric Variables and Derived Indices

1. Basic anthropometric measurements

Variable	Definition / Formula	Interpretation / Measurement Note
body mass	Total body mass of the participant, measured using a calibrated scale, usually reported in kilograms (kg).	Basic size and mass indicator.
height	Vertical distance from the floor to the vertex of the head while standing erect, usually reported in centimeters (cm).	Reference variable for body-size normalization.
arm span	Linear distance between the tips of the middle fingers when both arms are horizontally abducted at shoulder level.	Also commonly referred to as finger reach or wingspan.
sitting height	Vertical distance from the sitting surface to the vertex of the head in an upright seated posture.	Reflects trunk length contribution to total stature.
neck length	Linear or vertical distance between predefined cervical landmarks according to the measurement protocol.	The exact anatomical landmarks should be consistent with the study protocol.
hand breadth	Maximum transverse breadth of the hand, commonly measured across the metacarpal head region.	Reflects hand width.
biacromial breadth	Straight-line distance between the left and right acromion points.	Reflects shoulder width.
pelvic breadth	Transverse distance between the left and right pelvic bony landmarks, commonly iliac crest-related landmarks, according to the protocol.	Reflects pelvic skeletal width.
hip breadth	Maximum transverse breadth across the hip region at the level of the greatest lateral prominence.	Reflects hip and gluteal transverse width.
neck circumference	Circumference of the neck measured at a protocol-defined anatomical level.	Reflects neck girth.
chest circumference	Circumference of the thorax measured at a protocol-defined chest level, usually during normal breathing.	Reflects thoracic girth.
waist circumference	Circumference around the waist at the narrowest point or at a protocol-defined anatomical level.	Reflects abdominal girth.
hip circumference	Maximum circumference around the buttocks/hip region.	Reflects hip and gluteal girth.
upper-arm circumference (flexed)	Maximum circumference of the upper arm with the elbow flexed and the arm muscles contracted.	Reflects contracted upper-arm girth.
upper-arm circumference (relaxed)	Circumference of the upper arm measured while the arm is relaxed.	Reflects relaxed upper-arm girth.
forearm circumference	Maximum circumference of the forearm measured at the largest girth level.	Reflects forearm girth.
upper-limb length	Distance from the acromion point to the tip of the middle finger with the upper limb extended.	Reflects total upper-limb length.
upper-arm length	Distance from the shoulder/acromion landmark to the elbow landmark according to the protocol.	Reflects proximal upper-limb segment length.
forearm length	Distance from the elbow landmark to the wrist landmark according to the protocol.	Reflects distal upper-limb segment length.
palm length	Length of the palm segment measured from the wrist/palmar reference point to the distal palmar or metacarpal reference point according to the protocol.	Reflects palm segment length; landmarks should follow the original protocol.
hand length	Distance from the wrist reference point to the tip of the middle finger.	Reflects total hand length.
lower-limb length	Length of the lower limb. In this dataset, it is consistent with height minus sitting height.	Reflects lower-limb contribution to total stature.
thigh length	Distance from the hip or proximal thigh landmark to the knee landmark according to the protocol.	Reflects proximal lower-limb segment length.
shank length	Distance from the knee landmark to the ankle landmark according to the protocol.	Reflects distal lower-limb segment length.

Variable	Definition / Formula	Interpretation / Measurement Note
achilles tendon length	Distance from the calcaneal insertion region to the musculotendinous junction of the Achilles tendon along the posterior lower leg.	Reflects Achilles tendon relative morphology.
thigh circumference (contracted)	Maximum circumference of the thigh measured while the thigh muscles are contracted.	Reflects contracted thigh girth.
thigh circumference (relaxed)	Circumference of the thigh measured while the muscles are relaxed at a protocol-defined level.	Reflects relaxed thigh girth.
calf circumference (contracted)	Maximum circumference of the calf measured while the calf muscles are contracted.	Reflects contracted calf girth.
calf circumference (relaxed)	Maximum circumference of the calf measured while the calf muscles are relaxed.	Reflects relaxed calf girth.
ankle circumference	Circumference around the ankle at a protocol-defined anatomical level.	Reflects ankle girth and distal lower-limb skeletal/soft-tissue size.

2. Height-normalized ratios

Variable	Definition / Formula	Interpretation / Measurement Note
hand length-to-height ratio	hand length / height	Dimensionless index showing hand length relative to stature.
lower-limb length-to-height ratio	lower-limb length / height	Dimensionless index showing lower-limb proportion relative to stature.
upper-limb length-to-height ratio	upper-limb length / height	Dimensionless index showing upper-limb proportion relative to stature.
upper-arm length-to-height ratio	upper-arm length / height	Dimensionless index showing upper-arm length relative to stature.
forearm length-to-height ratio	forearm length / height	Dimensionless index showing forearm length relative to stature.
palm length-to-height ratio	palm length / height	Dimensionless index showing palm length relative to stature.
thigh length-to-height ratio	thigh length / height	Dimensionless index showing thigh length relative to stature.
shank length-to-height ratio	shank length / height	Dimensionless index showing shank length relative to stature.
achilles tendon length-to-height ratio	achilles tendon length / height	Dimensionless index showing Achilles tendon length relative to stature.
hand breadth-to-height ratio	hand breadth / height	Dimensionless index showing hand breadth relative to stature.
biacromial breadth-to-height ratio	biacromial breadth / height	Dimensionless index showing shoulder breadth relative to stature.
pelvic breadth-to-height ratio	pelvic breadth / height	Dimensionless index showing pelvic breadth relative to stature.
hip breadth-to-height ratio	hip breadth / height	Dimensionless index showing hip breadth relative to stature.
neck length-to-height ratio	neck length / height	Dimensionless index showing neck length relative to stature.
arm span-to-height ratio	arm span / height	Dimensionless index showing arm span or finger reach relative to stature.

3. Derived anthropometric indices

Variable	Definition / Formula	Interpretation / Measurement Note
Cormic Index	sitting height / height	Represents the proportion of trunk length relative to stature. If expressed as a percentage, multiply by

Variable	Definition / Formula	Interpretation / Measurement Note
		100.
Manouvrier's index	lower-limb length / sitting height	Represents the proportional relationship between lower-limb length and trunk length. If expressed as a percentage, multiply by 100.
brachial-antebrachial index	forearm length / upper-arm length	Represents the relative length of the forearm compared with the upper arm.
shank-to-thigh index	shank length / thigh length	Represents the relative length of the shank compared with the thigh.
hand shape index	hand breadth / hand length	Represents hand shape; higher values indicate a relatively broader hand.
thigh-to-calf girth ratio (contracted)	thigh circumference (contracted) / calf circumference (contracted)	Represents the relative contracted girth of the thigh compared with the calf.
thigh-to-calf girth ratio (relaxed)	thigh circumference (relaxed) / calf circumference (relaxed)	Represents the relative relaxed girth of the thigh compared with the calf.
achilles tendon length index	achilles tendon length / shank length	Represents Achilles tendon length relative to shank length.
calf morphology index (contracted)	calf circumference (contracted) / ankle circumference	Represents contracted calf girth relative to ankle girth.
calf morphology index (relaxed)	calf circumference (relaxed) / ankle circumference	Represents relaxed calf girth relative to ankle girth.
